# Whodunit? The Contribution of Interleukin (IL)-17/IL-22-Producing γδ T Cells, αβ T Cells, and Innate Lymphoid Cells to the Pathogenesis of Spondyloarthritis

**DOI:** 10.3389/fimmu.2018.00885

**Published:** 2018-04-25

**Authors:** Annika Reinhardt, Immo Prinz

**Affiliations:** Institute of Immunology, Hannover Medical School, Hannover, Germany

**Keywords:** interleukin-23, interleukin-17, γδ T cells, T_H_17 cells, innate lymphoid cells, spondyloarthritis

## Abstract

γδ T cells, αβ T cells, and innate lymphoid cells (ILCs) are capable of producing interleukin (IL)-17A, IL-17F, and IL-22. Among these three families of lymphocytes, it is emerging that γδ T cells are, at least in rodents, the main source of these key pro-inflammatory cytokines. γδ T cells were implicated in multiple inflammatory and autoimmune diseases, including psoriasis, experimental autoimmune encephalomyelitis and uveitis, colitis, and rheumatoid arthritis. Recent findings pointed toward a central role of γδ T cells in the pathogenesis of spondyloarthritis (SpA), a group of inflammatory rheumatic diseases affecting the axial skeleton. SpA primarily manifests as inflammation and new bone formation at the entheses, which are connecting tendons or ligaments with bone. In SpA patients, joint inflammation is frequently accompanied by extra-articular manifestations, such as inflammatory bowel disease or psoriasis. In humans, genome-wide association studies could link the IL-23/IL-17 cytokine axis to SpA. Accordingly, antibodies targeting IL-23/IL-17 for SpA treatment already showed promising results in clinical studies. However, the contribution of IL-17-producing γδ T cells to SpA pathogenesis is certainly not an open-and-shut case. Indeed, the cell types that are chiefly involved in local inflammation in human SpA still remain largely unclear. Some studies focusing on blood or synovium from SpA patients reported augmented IL-17-producing and IL-23 receptor-expressing γδ T cells, but other cell types might contribute as well. Here, we summarize the current understanding of how γδ T cells, αβ T cells, and ILCs contribute to the pathogenesis of human and experimental SpA.

## Introduction

Spondyloarthritis (SpA) encompasses a group of human rheumatic diseases that typically manifest as inflammation and new bone formation at axial joints, leading to severe lower back pain and impaired spinal mobility. Thereby, inflammation starts from entheses, the tendon to bone attachment sites. The family of SpA includes ankylosing spondylitis (AS), the SpA prototype (1), reactive arthritis, axial and undifferentiated SpA as well as psoriatic arthritis (PsA) and inflammatory bowel disease (IBD)-associated arthritis. Different SpA pathologies demonstrate similar disease patterns and similar genetic associations. First, the MHC class I molecule HLA-B27 was identified to confer susceptibility to SpA, and HLA-B27 is present in approximately 90% of AS patients in Europe (2). Over the last few years, more and more genome-wide association studies revealed a link between the interleukin (IL)-23/IL-17 axis and SpA susceptibility (3–6). Newly identified susceptibility genes comprise *IL-12B, IL-1R, CARD9, TYK2, STAT3*, and *IL-23R*, the gene encoding for the IL-23 receptor (IL-23R). The latter is particularly interesting, because single nucleotide polymorphisms in *IL-23R* were associated not only with AS (7) or PsA (8) but also with psoriasis (9) and IBD (10), hence pathologies that frequently accompany articular inflammation in SpA.

Nonsteroidal anti-inflammatory drugs and TNF inhibitors serve as first-line treatment for SpA. However, new treatment strategies emerged with the identification of the IL-23/IL-17 axis as putative key pathway associated with SpA. Most prominently, anti-IL-17A (receptor) treatment improved SpA disease symptoms (11–17). By contrast, IL-23 inhibition presented ambiguous results (18–21) (ClinicalTrials.gov number NCT02437162). If these drugs should completely replace old treatment modalities in the future, it still needs to be validated further (22–24).

Enthesitis (25), thus entheseal inflammation, represents a main characteristic of SpA. It was suggested that mechanical stress and local microdamage might initiate entheseal inflammation (26, 27), proposing the enthesis as primary lesion in SpA-associated joint inflammation (28–30). However, the link between host genetics, e.g., the IL-23/IL-17 axis, and local inflammation as well as new bone formation is not entirely clear. Strikingly, several SpA-focused studies suggested that the IL-23/IL-17 cytokine axis and innate immune activation might be of greater importance than classical autoreactivity of B or T cell receptors (6, 31, 32). Indeed, several albeit not all SpA patients demonstrated an increase in IL-23/IL-17 serum or synovial fluid levels (33–37). IL-17 cytokines are usually produced by lymphocytes, although earlier studies observed IL-17-producing mast cells (38), neutrophils, and myeloperoxidase-expressing cells (39) in SpA synovia. So, who does it? In the following, we summarize and discuss current data about human and experimental SpA and the three prime suspects of the IL-23/IL-17 axis: γδ T cells, αβ T cells, and innate lymphoid cells (ILCs).

### γδ T Cells

Although pre-committed effector γδ T cells represent a major source of IL-17/IL-22 under steady-state conditions in rodents (40–42), data reporting IL-17/IL-22-producing γδ T cells in healthy human individuals are rare (42–44). However, γδ T cells are clearly associated with different infections and tumors as well as autoinflammatory and autoimmune diseases in humans (45, 46). First studies suggesting a possible connection between γδ T cells and SpA were published approximately 30 years ago, just shortly after the discovery of γδ T cells (47, 48). By now, a number of studies demonstrated a decrease of γδ T cells in blood (49–51), while others showed that γδ T cells were frequently present in SpA patients’ synovial fluid (52, 53), suggesting that γδ T cells might play a role in disease induction and/or persistence in humans.

In fact, a direct association of γδ T cells and IL-17/IL-22 secretion in human SpA was first described by Kenna and colleagues, demonstrating an enrichment of IL-23R^+^ IL-17-producing γδ T cells in blood of AS patients (54). Strikingly, this phenotype was absent in rheumatoid arthritis patients (54), suggesting specific involvement of IL-17-producing γδ T cells in SpA pathogenesis rather than in arthritic inflammation in general. Along the same line, the analysis of tissue samples from enthesitis-related arthritis (55), reactive arthritis or undifferentiated SpA (56) as well as juvenile idiopathic arthritis (JIA) patients (57) revealed an increase in blood and synovial fluid IL-17-producing γδ T cells. Notably, such increased numbers of IL-17-producing γδ T cells might be driven by a defined arthritic cytokine environment (57). Although IL-23 certainly represents the main driver cytokine inducing enhanced IL-17 secretion by different cell types, also IL-9-driven expansion of IL-17-producing γδ T cells in PsA synovial fluid was recently demonstrated (58).

γδ T cells were implicated not only in SpA and related diseases in humans but also in mice. In various mouse models for non-autoimmune arthritis, including non-autoimmune antigen-induced arthritis (59), mannan-induced arthritis (60), or CFA-injected IFN-γ^−/−^ mice (61), γδ T cells were increased in numbers and were the main source of pathogenic IL-17 in inflamed tissues.

So, how do SpA-associated IL-17-producing γδ T cells get into inflamed sites in humans and mice? It is tempting to speculate that circulating and/or γδ T cells from distant tissues might leave their sites of origin and gather at the sites of crime, the inflamed joints. Accordingly, blood isopentenyl pyrophosphate-responsive Vγ9^+^ (53) or α4β7^+^ mucosal (γδ) T cells (52) might preferentially accumulate in JIA joints during an acute flare or a low acute-phase response, respectively. Likewise, CCR2^+^Vγ6^+^ IL-17-producing γδ T cells were recruited to joints by CCL2-inducing CD4^+^ T cells in arthritic *Il-1rn*^−/−^ mice (62).

However, there is more to be considered than migration of γδ T cells into inflamed tissues when trying to solve the case of idiopathic local entheseal inflammation in SpA. Applying an IL-23-dependent mouse model resembling inflammation-driven bone destruction (63) and most features of human SpA (64), tissue-resident IL-23R^+^RORγt^+^CD3^+^CD4^−^CD8^−^ lymphocytes were discovered in mouse entheses (64). Systemic IL-23 overexpression induced local inflammation in the enthesis by triggering resident IL-23R^+^RORγt^+^CD3^+^CD4^−^CD8^−^ lymphocytes to secrete IL-17 and IL-22, ultimately leading to IL-17-dependent enthesitis, IL-22-dependent bone remodeling as well as aortic root inflammation and psoriasis (64). Based on this study, we could recently demonstrate that Vγ6^+^ γδ T cells reside within mouse entheses, where they constitute the large majority of IL-23R^+^RORγt^+^CD3^+^CD4^−^CD8^−^ lymphocytes in steady state and increase in numbers during inflammation (65). Whether *Tcrd*^−/−^ mice would thus be protected from IL-23-dependent entheseal inflammation is still a matter of investigation. Notably, aging male DBA mice still develop severe enthesitis and new bone formation in the absence of γδ T cells (66). However, whether this phenotype results from entheseal γδ T cell redundancy or an increased presence of enthesis-resident lymphocytes other than γδ T cells that functionally refills their empty niche (67) still remains an open question.

**Figure 1:**
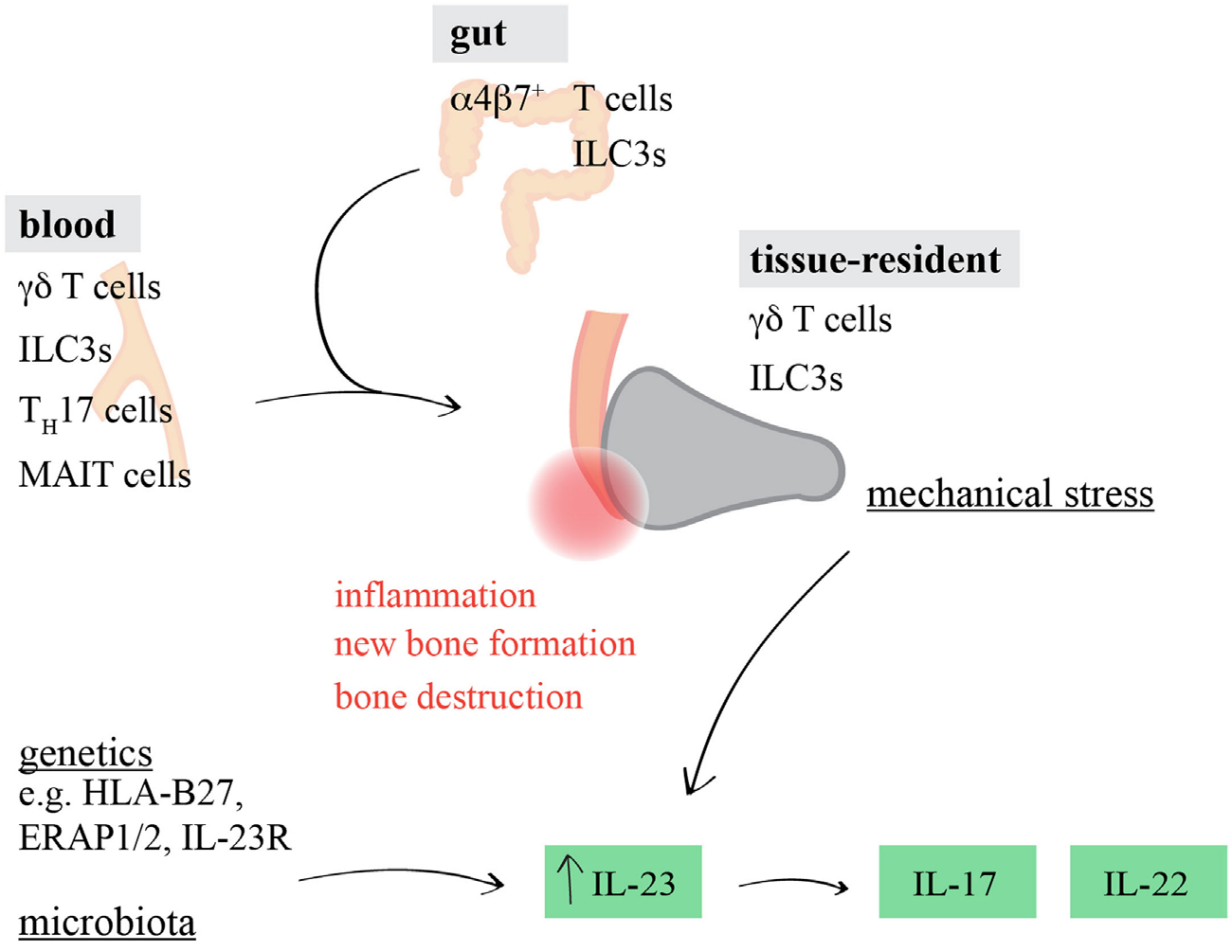
Involvement of interleukin (IL)-17/IL-22-producing lymphocytes in spondyloarthritis (SpA)-associated inflammation. Genetic and epigenetic predisposition, altered microbial composition, and entheseal microdamage can influence the induction and progression of tissue inflammation in SpA. In addition to tissue-resident γδ T cells and ILC3s, circulating and/or gut-derived γδ T cells, ILC3s, T_H_17 cells, or mucosal-associated invariant T (MAIT) cells might promote IL-23-driven joint inflammation by producing increased amounts of IL-17 and IL-22.

Strikingly, resident γδ T cells have just recently also been identified in human entheses (68). Thus, it appears likely that under steady-state conditions, entheseal γδ T cells reside in this very specific anatomical niche to control tissue homeostasis and possibly physiological bone remodeling after injury and exercise, while upon the elevation of IL-23 serum levels, they are driven to increase IL-17/IL-22 production and thus promote SpA (Figure 1).

### Innate Lymphoid cells

Innate lymphoid cells were identified around 10 years ago (69), but might have played important roles in joint inflammation even before adaptive immune cells developed over 450 million years ago. By now, it is clear that ILCs are crucially involved in the pathogenesis of a variety of inflammatory diseases, but also in tissue homeostasis (70, 71). For example, different ILC subsets were implicated in human and experimental rheumatic diseases (72). Recently, elevated numbers of ILC1s, ILC2s, and ILC3s were measured in blood samples from PsA patients, while only ILC3 numbers positively correlated with disease activity (73). However, circulating ILC3s in PsA patients displayed an immature phenotype, only produced moderate amounts of IL-17/IL-22, and did not express NKp44 (73). Interestingly, this further implies that final ILC3 maturation might only occur directly at the site of inflammation, presumably directly in the joints. However, as ILCs possess a high degree of plasticity (74), the transformation of inflammatory ILC1s or ILC2s into ILC3-like cells might also be possible (75, 76). In addition to an enrichment of NKp44^−^ ILC3s in PsA blood, NKp44^+^CCR6^+^ IL-17- (77) or GM-CSF-producing (78) ILC3s were abundantly present in SpA synovial fluid. Here, circulating NKp44^+^ILC3 numbers inversely correlated with disease activity (77), possibly resulting from the migration of circulating (immature) ILC3s to target tissues.

Association between SpA and intestinal inflammation is well established (79), and at least 50% of SpA patients suffer from (sub)clinical gut inflammation (80). A connection between ILCs and SpA-associated gut inflammation was described by different studies. In blood from entheropathic SpA patients, levels of IL-17-producing ILC3s were significantly higher as compared to IBD patients and healthy controls (81). While intestinal CD4^+^ T cells represented the main source of local IL-22 in Crohn’s disease patients, IL-22-producing NKp44^+^ ILCs were predominant in the gut of AS patients (82), highlighting differences between the etiology of IBD and SpA-associated intestinal inflammation. Surprisingly, particularly α4β7^+^IL-23R^+^ IL-17/IL-22-producing ILC3s were increased in gut, blood, bone marrow, and synovial fluid from AS patients with intestinal inflammation, suggesting that gut-derived functionally mature ILC3s might emigrate from intestinal tissues to α4β7 ligand-expressing joints, promoting local SpA-associated inflammation (83). SpA-associated α4β7^+^ synovial T cells were also already described before (84). Based on these results, ILC3s were proposed to function as “cytokine shuttles from gut” to extra-intestinal tissues (85). Strikingly, IL-17/IL-22-producing ILC3s appeared to be RORγt^−^ but Tbet^+^ (83), possibly reflecting a particular developmental stage (86).

Although tissue residency is not well established for human ILCs, there is good evidence that ILCs in mice are generally tissue-resident (87, 88). Thus, the migration of gut-derived ILCs into SpA joints appears surprising at first glance. However, photoconversion experiments in mice could reveal CCR7-dependent ILC3 migration from gut to mesenteric lymph nodes (89), supporting the notion that intestinal ILCs can, at least to some extent, traffic to distant sites. The expression of a particular chemokine/cytokine signature, homing receptors, and respective ligands might promote their traveling. For instance, NKp44 ligand was shown to be expressed by chondrocytes, even in non-inflamed joints (90).

The migration of peripheral ILC3s into inflamed tissues in the context of SpA appears to be an interesting hint. However, the presence of resident NKp44^+^ ILC3s in non-inflamed human spinal entheses has just been reported (68). Further, that study demonstrated that, in consistence with mouse entheseal tissues (64, 65), human entheses responded to stimulation with IL-23/IL-1β by increasing IL-17/IL-22 production (68). Thus, in addition to γδ T cells, enthesis-resident ILC3s represent another candidate of innate lymphocytes that might be involved in the induction and/or progression of SpA (Figure 1).

### αβ T Cells

Although recent studies point toward a chief contribution of innate lymphocytes to SpA, it is inevitable to include αβ T cells into the inner circle of prime suspects.

The “arthritogenic peptide theory” suggested that CD8^+^ T cells specific for HLA-B27-presented peptides might be involved in SpA disease pathogenesis (91, 92). Some immunohistological analyses indeed demonstrated that CD8^+^ T cells were predominant in human entheseal infiltrates (93, 94). However, studies in an experimental SpA model, HLA-B27-transgenic rats, did not support this theory (95, 96). Notably, HLA-B27 molecules can also be recognized as B27 β2-microglobulin-free heavy chains by killer immunoglobulin-like receptors (KIRs) (97, 98). HLA-B27/KIR3DL2 binding can induce RORγt expression in CD4^+^ αβ T cells and thus a T helper 17 (T_H_17) cell phenotype (99). Accordingly, although not confirmed in early axial SpA (100), KIR3DL2^+^ T_H_17 cells were increased in AS patients, suggesting that these cells might represent a therapeutic target for SpA treatment (101). HLA-B27 also appears to be associated with dysbiosis (102–105), possibly resulting from HLA-B27 misfolding-induced (106, 107) upregulation of the IL-23/IL-17 axis (108), innate immune activation, and intestinal T_H_17 cell expansion (109) early in life (110).

In fact, several albeit not all (39, 54, 111) SpA studies observed increased amounts of T_H_17 cells in blood and/or synovial fluid (100, 112–117), possibly in a sex-dependent manner (118). While IL-17 is the most representative cytokine ascribed to T_H_17 cells, GM-CSF-producing T_H_17 cells were also elevated in SpA patients (78). However, the increase in GM-CSF-producing lymphocytes was not specific for T_H_17 cells, as IL-17-producing CD8^+^ T cells, γδ T cells, and ILC3s co-producing GM-CSF were similarly expanded (78).

Supposing that increased numbers of T_H_17 cells promote SpA—how to keep these cells in check? In fact, miR-10b-5p (119) and IL-10-producing B cells (120) were recently proposed as putative negative regulators trying to control T_H_17 cells from SpA patients. Although increases in regulatory T cells in gut (121), blood, and synovial fluid (122, 123) from SpA patients also hinted toward an unsuccessful reaction to suppress autoinflammation, these cells might be functionally defect (124), demonstrating an imbalance in IL-10/IL-17 production (125).

Finally, unconventional/innate IL-17/IL-22-producing αβ T cell subsets might be associated with SpA. While neither human nor experimental SpA-associated IL-17-producing invariant natural killer T cells were identified so far, IL-17-producing CD8^+^ T cells (78, 126) or mucosal-associated invariant T (MAIT) cells (127, 128) were described. Surprisingly, increased IL-17 production by MAIT cells derived from AS patients was IL-23-independent, but rather promoted by IL-7 (128)—similar findings were recently reported in multiple sclerosis patients (129). Indeed, *IL7R* polymorphisms are associated with AS (4), and it was proposed that mechanical stress-induced IL-7 secretion by synovial fibroblasts could induce MAIT cell activation and thus IL-17 secretion during SpA pathogenesis (130). However, enthesis-resident MAIT cells have not been described so far.

Although seemingly plenty of studies described an association of T_H_17 cells and SpA, CD4^+^ cell depletion did not protect from IL-23-dependent inflammation (64). Along the same line, aging male DBA *Tcrb*^−/−^ mice did still develop enthesitis and new bone formation (66), and γδ T cells, but not αβ T cells, dominated among pathogenic IL-17-producing enthesis-resident lymphocytes in mice (65). Together, this indicates that T_H_17 cells might rather not mediate the first line of action when local entheseal immune cells are provoked by the various environmental triggers, such as mechanical stress, dysbiosis, or genetic and epigenetic predisposition (131) (Figure 1).

## Concluding Remarks: Whodunit?

After lining up the usual and a few unusual suspects, it seems that although strong arguments point toward an important contribution of the IL-23/IL-17 axis mediating SpA, it still remains difficult to pinpoint which of the above-described cell types are major players (132). Overall, recent studies collectively favor innate and innate-like immune cell involvement rather than adaptive T cells (6, 31, 32). As opposed to conventional B and T cells, innate and innate-like lymphocytes are commonly enriched in non-lymphoid tissues, and thus association with autoinflammatory diseases affecting particular tissues appears feasible (133). In this respect, it is worth considering the differential effects that genetics and environmental factors might elicit in innate versus adaptive immune traits (134).

Still, the relative contributions of tissue-resident cells, i.e., γδ T cells and ILC3s, versus recruited cells to SpA pathologies are not entirely clear. The “mechanical stress and entheseal microdamage hypothesis” (26, 27) supports the idea of inflammation-promoting enthesis-resident cells. In this regard, one might hypothesize that mechanical stress triggers resident immune cell activation—either directly or indirectly *via* stromal cell activation. Indeed, mechanical stress was shown to support enthesitis and new bone formation in TNF^ΔARE^ and aging male DBA mice, whereas remarkable experiments involving hind limb unloading significantly reduced disease symptoms (135). Since human tissues are generally difficult to obtain, various animal studies experimentally addressed immune pathways associated with SpA. However, it should be noted that many animal models only work in a specific genetic background, and vast differences exist between individual SpA models. While some models strongly depend on IL-23, others are based on TNF dysregulation (136, 137). Consequently, experimental data might be controversial: while Rag2^−/−^ mice did not develop pathologies upon IL-23 overexpression (64), arguing against a role for innate lymphocytes in disease induction, enthesitis in Rag1^−/−^ TNF^ΔARE^ mice was unaffected (135).

In the human system, many traits originate from analysis of circulating immune cell populations. However, such data remain inherently difficult to interpret: an increase in a particular circulating cell population does not unequivocally suggest their increased migration to joint tissues, while a decrease cannot unambiguously imply these cells already relocated from blood into distant sites. And why should otherwise tissue-resident ILC3s, T_H_17 cells, MAIT cells, or γδ T cells, leave the intestine and migrate into axial sites and distant tissues? Indeed, increased levels of CCL20, the chemokine attracting CCR6^+^ cells, were detected in SpA joints, albeit not as prominent as in rheumatoid arthritis (138). Altered gut epithelial and vascular barrier integrity in SpA patients might further promote intestinal immune cell emigration (139). Importantly, the trafficking of intestinal IL-17-producing γδ T cells to an entirely different tissue, the leptomeninges, was described in a mouse model for stroke (140, 141). Notably, a gut/joint axis in SpA might also exist for antigen-presenting cells carrying bacterial antigens from gut to axial sites, thus contributing to the induction of a local immune response (142). Whether SpA-associated joint-infiltrating lymphocytes enter tissues as already activated and functionally mature cells also remains an open question. Relatedly, CXCL4 was recently identified as a novel potent inducer of human T_H_17 cells enriched in PsA joints, and CXCL4 levels also positively correlated with disease severity, thus suggesting a CXCL4-driven local boost of T_H_17 cells (143).

In conclusion, there is growing evidence that innate and tissue-resident IL-17-producing γδ T cells and perhaps also ILC3s might be locally primed by genetic and epigenetic predisposition, mechanical stress as well as by increased systemic inflammation caused by intestinal dysbiosis. However, future studies will need to elaborate prevailing theories about the SpA-associated sequence of events. No matter whether ILC3s, γδ T cells, or any other innate lymphocytes primarily promote inflammation in SpA, in the end, the pathogenic action of all these cell types can be collectively targeted *via* the IL-23/IL-17 axis.

## Author Contributions

All the authors listed have made a substantial, direct, and intellectual contribution to the work and approved it for publication.

## Conflict of Interest Statement

The authors declare that the research was conducted in the absence of any commercial or financial relationships that could be construed as a potential conflict of interest. The reviewer GG and handling Editor declared their shared affiliation.
